# Phenolic Compounds Characterization of *Caryocar brasiliense* Peel with Potential Antioxidant Activity

**DOI:** 10.3390/plants13152016

**Published:** 2024-07-23

**Authors:** Júlio Onésio Ferreira Melo, Beatriz Conchinhas, António Eduardo Baptista Leitão, Ana Luiza Coeli Cruz Ramos, Isabel Maria Nunes de Sousa, Ricardo Manuel de Seixas Boavida Ferreira, Ana Cristina Ribeiro, Paula Batista-Santos

**Affiliations:** 1 Departamento Ciências Exatas e Biológicas, Universidade Federal de São João Del-Rei (UFSJ), Sete Lagoas 35701-970, MG, Brazil; 2 Tropical College of the University of Lisbon—CTROP-ULisboa, Alameda da Universidade—Cidade Universitária, 11649-004 Lisbon, Portugal; 3 Forest Research Centre, Associate Laboratory TERRA, School of Agriculture, University of Lisbon, Tapada da Ajuda, 1349-017 Lisbon, Portugal; 4 Departamento de Alimentos, Faculdade de Farmácia, Universidade Federal de Minas Gerais (UFMG), Belo Horizonte 31270-901, MG, Brazil; 5 LEAF—Linking Landscape, Environment, Agriculture and Food—Research Center, Instituto Superior de Agronomia, Universidade de Lisboa, Tapada da Ajuda, 1349-017 Lisbon, Portugal; 6 Faculdade Farmácia, Universidade de Lisboa, Av. Prof. Gama Pinto, 1649-003 Lisbon, Portugal

**Keywords:** pequi, *Caryocar brasiliense*, phenolic compounds, antioxidant activity, flavonoids, TPC, DPPH, FRAP, ABTS, Brazilian Cerrado

## Abstract

The pequi (*Caryocar brasiliense*) fruit peel, despite being frequently discarded, has a high content of bioactive compounds, and therefore has a high nutritional value. The present study aimed to explore the bioactivities in the pequi peel, particularly their potential health benefits at the level of antioxidant activity. The exploitation of this fruit could also present significant economic benefits and applications of pequi by-products would represent a reduction in waste, having a positive impact on the environment. Phenolic compounds present in the pequi exocarp and external mesocarp were identified by paper spray mass spectrometry (PS-MS) and quantified by HPLC. The total phenolic content (TPC) along with the amount of 2,2-diphenyl-1-picrylhydrazyl (DPPH), Ferric Reducing Antioxidant Power (FRAP), and the amount of 2,2′-azino-bis(3-ethylbenzothiazoline-6-sulphonic acid (ABTS) were also determined in peel extracts. Epicatechin was the most abundant phenolic compound found, followed by the caffeic, salicylic, and gallic acids. In addition, fingerprinting revealed compounds related to several beneficial health effects. In short, the results obtained were encouraging for potential applications of pequi peel in the field of functional foods.

## 1. Introduction

The Cerrado region in Brazil is the world’s richest tropical savanna in terms of biodiversity and the second most extensive biome in South America [1]. Plants from the Cerrado have been receiving increased attention as a source of bioactive compounds [2] which have applications in the pharmaceutical and food industries. The pequi tree, in particular, stands out in this regard.

The pequizeiro (*Caryocar brasiliense*) is a medium-sized tree native to the Brazilian Cerrado, belonging to the Caryocaraceae family and the genus *Caryocar* [3,4]. Among the species of this genus, *Caryocar brasiliense* Camb is the most abundant in Brazil [4]. The pequizeiro fruits, also called pequi, are of significant cultural and economic importance and are commonly used by the native population and exploited through extractivism [3,5]. The fruits are produced between November and March and are appreciated for their pleasant peculiarities of color, aroma, and flavor, not only in traditional Brazilian dishes but also for oil extraction and liqueur production [6]. In traditional medicine, it is used to treat flu and liver and stomach diseases and as a tonic and aphrodisiac [7].

**Figure 1:**
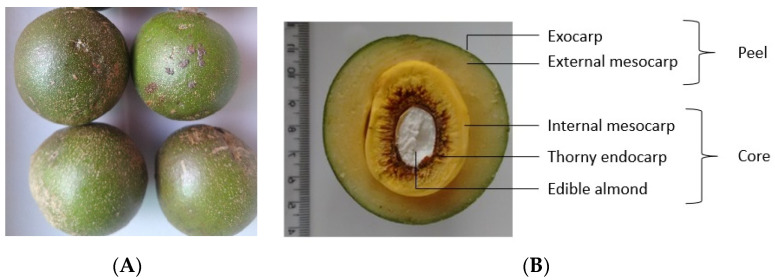
Overview of pequi (*Caryocar brasiliense* Camb.) fruit: (**A**) whole fruits and (**B**) longitudinal cut of the fruit and identification of its components. Adapted from [10,11].

The fruit of the pequizeiro tree is a drupe (Figure 1) and the edible pulp is the most valued part of the fruit. It is characterized by a high number of natural compounds of interest, such as fatty acids, dietary fiber, zinc, magnesium, and polyphenols which are related to antioxidant activity and the neutralization of reactive oxygen species [8,9]. This pulp consists of a thick layer corresponding to the internal mesocarp, enclosing the seed covered by a thorny endocarp. It may contain up to three or four seeds, all surrounded by the external mesocarp and a green exocarp, known as the peel [10,11].

In 2020, the pequi production was approximately 63,500 tons [12].

Pequi peel accounts for approximately 80% of the total fruit mass and is usually discarded as agro-industrial waste, demonstrating the amount of waste generated. According to the literature, the epicarp flour and external mesocarp of pequi are rich in total dietary fiber and carbohydrates, ashes, calcium, copper magnesium, and manganese [13].

Furthermore, studies show that pequi contains compounds that exhibit antioxidant properties [7,14]. Antioxidants are substances known to delay the onset of the oxidative process. They can be categorized into synthetic antioxidants or natural antioxidants, such as bioactive phenolic compounds [15]. There has been a growing demand for antioxidants in the pharmaceutical, cosmetic, and food industries.

Phenolic compounds are plant substances characterized by an aromatic ring bearing one or more hydroxyl groups, known for their antioxidant properties and for playing a crucial role in immune-defense responses, reducing the risk of diseases such as cancer and diabetes [16]. Studies have demonstrated that pequi pericarp flour contains bioactive compounds with high bioaccessibilities, overcoming one of the main limitations affecting their beneficial effects, thereby making them suitable for use as a food ingredient [17]. Additionally, natural antioxidants offer the advantage of high consumer acceptance and may represent a preferable option for slowing the number of oxidative modifications made to food and reducing age-related health risks [18].

Recent studies indicate that the amounts of phenolic compounds found in pequi peel are significantly higher compared to the small amounts present in other fruit tissues, such as the pulp. However, it is also suggested that the changes in phenolic compounds during gastrointestinal digestion, resulting from interactions with other components and modifications to their chemical structures, can affect their bioaccessibility. Investigating the bioaccessibility of polyphenols is crucial because compounds released from food during digestion are potentially more bioavailable and could offer beneficial effects [19]. Only a few applications of pequi peel have been described in the literature so far, namely its use in the development of cookies and breads [20]. Therefore, exploiting this fruit residue could yield significant economic benefits, while reducing waste would have a positive environmental impact. In this way, this study aimed to search for bioactive compounds in *Caryocar brasiliense* Camb. peel with potential antioxidant activity to expand our knowledge about the little-explored parts of this fruit.

## 2. Results and Discussion

The Brazilian endemic flora is a reservoir of multiple bioactive compounds, and in this context, it is important to explore the antioxidant potential of the pequi fruit by analyzing the peel extracts.

### 2.1. Total Phenolic Content (TPC) and Antioxidant Activity (DPPH, FRAP, and ABTS)

The results for total phenolic content, along with the values for the antioxidant activity obtained from the FRAP, DPPH, and ABTS assays, are presented in Table 1 (the calibration curves are provided in the Supplementary Material).

The samples showed a TPC value around 204 mg GAE/g dry mass. The antioxidant activity was quantified as the TEAC (Trolox Equivalent Antioxidant Capacity), presenting values of 4025 µmol Trolox/g dry mass for the FRAP method, 2600 µmol Trolox/g dry mass for DPPH, and 12,400 µmol Trolox/g dry mass for ABTS.

As shown, the TEAC results suggest a good antioxidant capacity. Adjusting the values to the dry mass, by defining the fruits’ water content as being around 75%, allowed for comparisons to be made with other fruits and vegetables previously analyzed, from which the pequi peel extracts stood out with much higher values.

Thaipong et al. [21] reported values for the total phenolics in guava fruits (water content of about 80%) for around 15 mg GAE/g dry mass, and showed a considerably high antioxidant activity of methanol extracts determined by DPPH and FRAP assays of 126 µmol Trolox/g dry mass and 130 µmol Trolox/g dry mass, respectively. Other studies of antioxidant activity and phenolic content in the peel of tropical fruits from Yucatan, Mexico, presented ABTS and DPPH values of approximately 30 µmol Trolox/g and 16 µmol Trolox/g dry mass, respectively [22]. For peel extracts, Monteiro et al. [18] also reported high values of 78.58 mg GAE/g (dry mass) for polyphenol content. Additionally, Roesler et al. [23] determined the polyphenols in ethanolic extracts of pequi peel and reported even higher values (209.37 mg GAE/g dry mass), which are very similar to those obtained in the present study.

A recent study performed by Braga et al. [24] with ethanolic pequi peel extracts reported much lower total phenolic contents (696.91 mg of GAE/100 g dry mass) and antioxidant activity measured by the DPPH method (30.1 µmol of Trolox/g dry mass). The different procedures used in the extractions (type and proportion of solvents) may explain the high amplitude of total phenolic contents and antioxidant capacity in pequi [25], and according to Leão and collaborators [14], extraction processes using methanol–acetone are more efficient than other processes. Furthermore, factors such as geographic location, soil, and climate can influence the characteristics of fruit metabolites [17].

### 2.2. Paper Spray Mass Spectrometry

The secondary metabolites present in plant species may have bioactive activity. They are usually present in foods in small quantities and are considered to be vital non-nutritional ingredients for maintaining human health [26]. Paper spray mass spectrometry (PS-MS) is a technique that uses ionization at room temperature. This technique has gained attention in recent years because it is highly sensitive, provides quick results, and can analyze compounds present in complex mixtures such as foods [27,28].

**Figure 2:**
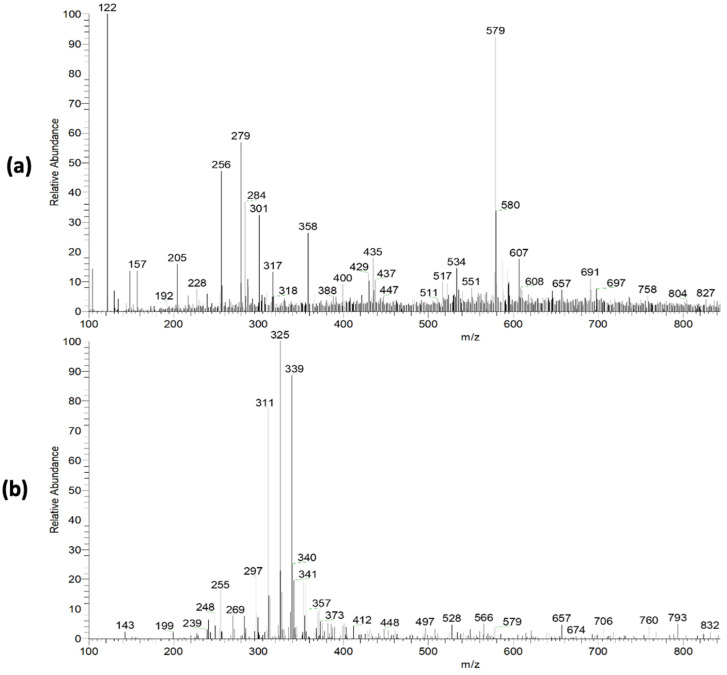
Mass spectra obtained in full scan mode with positive (**a**) and negative (**b**) ionization mode for pequi peel extract.

The pequi extract mass spectra (PS-MS) in positive (Figure 2a) and negative (Figure 2b) ionization mode are shown in Figure 2. For the pequi peel extract, seven compounds were found in the positive mode and 12 compounds in the negative mode (Table 2) of ionization, belonging to the classes of phenolic compounds, flavonoids, benzoic acid derivatives, sugar, and phenylpropanoids.

The flavonoid class was predominant in the present study, representing 42.86% of the proposed metabolites. Many of these compounds found in polyphenol occurrences are known for their antioxidant properties [38], corroborating the capacity assessment results observed for pequi extracts. These metabolites may be responsible for this capacity, such as the quercetin and quercetin derivatives [M − H]^−^ *m*/*z* = 433), the presence of which was previously associated with antioxidant activity for *C. brasiliense* fruits [39]. Furthermore, according to Harun-Or-Rashid et al. [40], vegetable polyphenols and flavonoids with antioxidant properties also have potential antibacterial activity.

The ion of *m*/*z* 301 ([M − H]^−^), associated with ellagic acid, is associated with antimutagenic, antioxidant, neuroprotective, and anti-inflammatory activity, being reported in the literature for several popular fruits, such as grapes and strawberries [41]. Santos et al. [17], observed several of these compounds in a product obtained from parts of the pequi fruit, such as chrysoeriol [M − H]^+^ *m*/*z* = 301), rhamnetin [M − H]^+^ *m*/*z* = 317), luteolin [M − H]^−^ *m*/*z* = 285), hexoside p-coumaric acid [M − H]^−^ *m*/*z* =325). These authors reinforce the association of these compounds with various pharmacological and health-beneficial activities, such as antibacterial, antifungal, anti-inflammatory, and anticancer, and a high antioxidant capacity [17]. Furthermore, Braga and collaborators [24] showed that reducing ROS levels by using pequi extract may also decrease LDL oxidation. In this way, it is possible to associate the presence of these compounds with the antioxidant capacity of the pequi extracts reported in the present study. Given the above, it is possible to highlight the high functional potential of the extract from non-conventionally used parts of the fruit, thus contributing to the use of food waste, and reducing the environmental impact.

### 2.3. Quantification of Phenolic Compounds—HPLC

**Figure 3:**
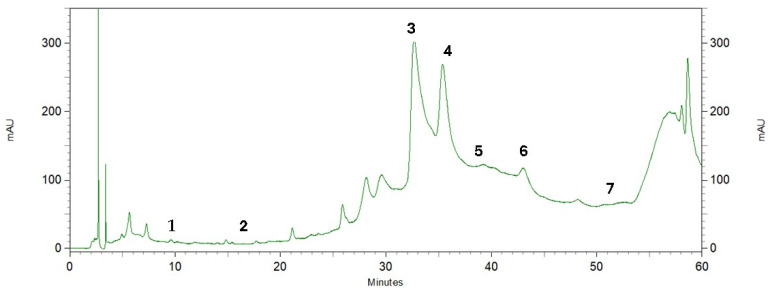
Chromatographic profile by HPLC of pequi peel extracts recorded at 280 nm. Phenolic compounds: (1) gallic acid, (2) protocatechuic acid, (3) caffeic acid, (4) epicatechin, (5) vanillin, (6) salicylic acid, (7) ferulic acid.

**Figure 4:**
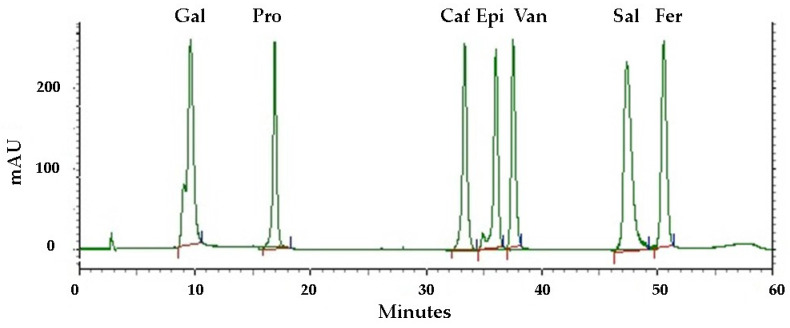
HPLC chromatograms of standards tested for comparison recorded at 280 nm. Phenolic compounds: gallic acid (Gal), protocatechuic acid (Pro), caffeic acid (Caf), epicatechin (Epi), vanillin (Van), salicylic acid (Sal), ferulic acid (Fer). The red and the blue lines represent the baseline and the limits, respectively, for the integration of the respective areas for quantifying different compounds.

Phenolic compounds are found in a variety of plant materials as secondary metabolites with anticancer, antibacterial, and antioxidant activities [42]. The HPLC analysis of the pequi peel extracts produced the chromatogram shown in Figure 3. Four phenolic compounds were identified by comparing their retention time with the peaks from seven standards tested (Figure 4) and were detected at the wavelength of 280 nm: gallic acid, protocatechuic, caffeic acid, epicatechin, vanillin, salicylic and ferulic acid. Epicatechin was the most abundant compound in the peel, with an overall mean content of 7.36 mg/g dry mass, followed by caffeic acid with approximately 4.31 mg/g dry mass (Table 3). Salicylic and gallic acid were found in smaller amounts. There were other peaks shown in the chromatogram for which substances were not identified due to a lack of phenolic standards for comparison.

In a recent qualitative study performed by Braga et al. [24], gallic, protocatechuic, gentisic, caffeic, p-coumaric, vanillic, and ellagic acids, as well as catechin, quercetine, epicatechin, rutin, naringenin, luteolin, and kaempferol, were also identified in the pequi peel extracts by HPLC-HRMS. When compared to several other fruits analyzed through HPLC [43], the pequi peel extracts presented much higher amounts of phenolic compounds, mainly epicatechin, caffeic acid, and salicylic acid. These results are corroborated by the results of Santos et al. [44], who have also identified salicylic acid derivates in *Caryocar brasiliense* Camb. Peel by CG-MS.

The function of phenolic compounds is mostly related to their antioxidant action, which is often connected with the prevention of several diseases [45]. Some of the phenolic compounds that were identified in pequi fruits, namely gallic acid and epicatechin, are tannins, and present high antioxidant capacity and the ability to eliminate of free radicals, therefore validating the medicinal properties of pequi according to popular use [46]. As it was observed that the form of extraction can influence the identification and quantification of compounds in addition to their activity, the results of the present study can point to future research that evaluates the efficiency of extraction methods.

## 3. Materials and Methods

### 3.1. Reagents and Chemicals

All chemicals and solvents used were of reagent or HPLC grade. For the paper spray mass spectrometry assay, methanol was purchased from Tedia, Fairfield, OH, USA. For the other assays, methanol was supplied by Carlo Erba (Cornaredo, Milan, Italy). Acetic acid (99–100%, HPLC grade) was obtained from Chem-Lab (Zedelgem, Belgium). Folin–Ciocalteu reagent was supplied by Sigma-Aldrich (St. Louis, MO, USA). Water was obtained using a Milli-Q water purification system (Millipore, Bedford, MA, USA). The standard stock solutions were obtained from Sigma Aldrich (St. Louis, MO, USA). All other substances were acquired from Sigma-Aldrich, Lisbon, Portugal.

### 3.2. Plant Material

The ripe pequi fruits were collected on the ground in Sete Lagoas (latitude 19°28′48″ and longitude 44°11′57″), state of Minas Gerais (Brazil). The samples are registered in the Sistema Nacional de Gestão do Patrimônio Genético e do Conhecimento Tradicional Associado—SisGen under code A7832EC. The fruits were washed, brought to Portugal, and stored at −80 °C. The fruits were manually cut to separate the endocarps from the intact peel (external mesocarp and exocarp) which was then processed as follows.

### 3.3. Preparation of Extracts

#### 3.3.1. Extracts for Total Phenolic Content and Antioxidant Activity Assay

The peel was minced using a food processor (Moulinex-123 A320R1, Lisbon, Portugal). The sample was ground with the 50% (*v*/*v*) methanol (solvent at a ratio of 1:4 (*m*/*v*) in a pestle and mortar and mixed using a vortex mixer for 30 s, followed by agitation for 1 h at 4 °C. The sample was centrifuged at 18,000× *g* for 15 min at 4 °C, the supernatant was collected, and 70% (*v*/*v*) acetone solvent at the same ratio (1:4, *m*/*v*) was added to the precipitate (pellet). The solution with acetone was mixed using a vortex mixer for 30 s and then centrifuged at 18,000× *g* for 15 min at 4 °C. The obtained supernatant was combined with the previous and the total volume was increased to a final ratio of 1:20 (*m*/*v*) with MilliQ water.

#### 3.3.2. Extracts for Paper Spray Mass Spectrometry Assay

Pequi peel extracts were obtained in a 1:8 (*w*/*v*) ratio. The peel was crushed and homogenized with an analytical mill (IKA—A11 basic). Then, 1.0 g of the previously homogenized sample was weighed, and 8 mL of methanol was added. The sample was vortexed for 30 min and kept at rest for 24 h under refrigeration. Subsequently, the supernatant was transferred to microtubes (2 mL), and the extracts were stored at freezing temperature until PS-MS analysis.

#### 3.3.3. Extracts for High-Performance Liquid Chromatography Assay

The pequi peel was minced into small pieces. The phenolic content was extracted using 70% (*v*/*v*) methanol as solvent at the ratio 1:4 (*m*/*v*). The extraction was performed by grinding the sample directly with the solvent followed by stirring for 1 h at 4 °C.

The extracts were centrifuged at 18,000× *g* for 15 min at 4 °C, and the supernatants were collected and stored at −80 °C until further analysis. Three replicates were carried out.

### 3.4. Quantification of Total Phenolic Content and Antioxidant Activity

#### 3.4.1. Total Phenolic Content Assay

This method is based on the reduction of the Folin–Ciocalteu reagent in the presence of phenolics, which causes the absorbance measured at 725 nm to increase linearly with the concentration of phenolics in the solution. An oxidation/reduction reaction occurs, with the phenolic group being oxidized and the reagent being reduced [47].

The total phenolic content of the samples was determined according to Heredia and Cisneros-Zevallos [48] and Hillis and Swain [49] using 150 µL of the diluted sample (1:40 in acetone 70%, *v*/*v*) mixed with 150 µL of Folin–Ciocalteu reagent and 2.4 mL of MilliQ water. The reaction was interrupted with the addition of 300 µL of 1 M sodium carbonate and the samples were maintained in the dark for 2 h prior reading at 725 nm in spectrophotometer using a blank prepared with methanol as a control.

The samples were analyzed in triplicate and the total phenolic content was established using a standard curve of the equivalent of gallic acid and expressed in mg GAE/g dry mass.

#### 3.4.2. DPPH Assay

The DPPH assay was used to predict the antiradical activities of antioxidants. In its radical form, DPPH is reduced in the presence of an antioxidant molecule altering its previous violet color in methanol and giving rise to a yellow-colored solution, which produces a decrease in absorbance at 515 nm. This means that solutions with a higher antioxidant activity become more yellow [50].

This method was performed according to Brand-Williams et al. [51]. The DPPH solution used was prepared with DPPH reagent diluted in methanol until 0.98 units of absorbance at 515 nm was reached. The sample was diluted to 1:100 in acetone 70% (*v*/*v*) and 100 µL was added to the DPPH solution (3.9 mL). The reaction occurred for 40 min, and the samples were read at 515 nm, using a spectrophotometer (Agilent Technologies Cary 100 UV-Vis, Santa Clara, CA, USA).

#### 3.4.3. FRAP Assay

The FRAP assay determines the ferric-reducing antioxidant power of samples through ferric-to-ferrous ion reduction at low pH, which causes a colored ferrous-tripyridyltriazine complex to form. To obtain the results from this method, the absorbance of the test reaction mixtures is then measured and compared with the absorbance in a solution containing ferrous ions in known concentration.

The FRAP test followed the procedure of Benzie and Strain [52]. For this method the sample was diluted 1:200 in acetone 70% (*v*/*v*). The diluted sample (90 µL) was mixed with the FRAP solution (2.7 mL) and 270 µL of MilliQ water. The samples were then warmed in a water bath at 37 °C for 30 min and read at 595 nm.

#### 3.4.4. ABTS Assay

Antioxidant activity was quantified using ABTS solution (2,2′-azino-bis(3-ethylbenzothiazoline-6-sulphonic acid)) following the method described by Re et al. [53] and Rufino et al. [50] ABTS with potassium persulfate is reduced in the presence of hydrogen-donating antioxidants. This assay measures their relative ability to scavenge ABTS, and consequently, the antioxidant activity of the sample. The radical ABTS solution was prepared with 88 µL of potassium persulfate (37.8 g/L) mixed with 5 mL of ABTS stock solution (3.8 g/L) and placed in the dark for 16 h. The obtained ABTS solution was diluted in ethanol until an absorbance value of approximately 0.70 was read at 734 nm. The extract was diluted 1:100 in acetone 70% (*v*/*v*), and 30 µL of the solution was mixed with 3 mL of ABTS solution and incubated for 6 min. Then, the absorbance was measured at 734 nm using ethanol as a blank for the control.

The antioxidant activity (DPPH, FRAP and ABTS) was determined using standard calibration curves prepared with Trolox (6-Hydroxy-2,5,7,8-tetramethylchroman-2-carboxylic acid) and the results were expressed as Trolox Equivalent Antioxidant Capacity [TEAC (µmol Trolox/g dry mass)]. For each determination method, the samples were analyzed in triplicate.

### 3.5. Paper Spray Mass Spectrometry

The pequi peel extract was analyzed using mass spectrometry with ambient ionization using paper spray. To this end, the following instrumental conditions were used in the Thermo LCQ-Fleet mass spectrometer (ThermoScientific, San Jose, CA, USA). Capillary temperature, 275 °C; capillary voltage, 40 V; tube lens voltage, 120 V; voltage applied to the paper, 4.5 kV, where two μL of extract and 40 μL of methanol were also applied. Mass spectra were obtained from 100 to 1000 *m*/*z* in positive (+4.5 kV) and negative (−3.5 kV) ionization modes.

A subsequent fragmentation was carried out to obtain the ions’ fragmentation profile for a subsequent attempt to identify the metabolites. Data were extracted by Xcalibur software version 2.1 (Thermo Scientific, San Jose, CA, USA) processed using spreadsheet software (Excel, 2020, Microsoft, Redmond, WA, USA). The identification attempt was carried out by comparing the experimental fragmentation patterns with those described in the literature [54,55].

### 3.6. Quantification of Phenolic Compounds by HPLC

The pequi extracts were filtered using a 0.45 μm Millex nylon membrane (Millipore) before being injected into the chromatographic system. The solvents for the mobile phases were prepared using acetic acid in water (2%) for solvent A and methanol, water, and acetic acid (70:28:2, *v*/*v*/*v*) for solvent B. The analyses were performed using a Beckman System Gold HPLC equipment, with a Spherisorb ODS2 column (4.6 × 250 mm, Waters), and detection was carried out at 280 nm with a total run time of 60 min, column temperature of 20 °C, flow of 1.25 mL/min, and an injection volume of 20 μL in a gradient program until the end of the run, following that devised by Guedes et al. [46]: 100% solvent A from 0 to 5 min, 70% of solvent A from 5 to 25 min, 60% of solvent A from 25 to 45 min, 55% of solvent A from 43 to 50 min and 0% of solvent A for 10 min. Solvent A was increased to 100% to balance the column, followed by equilibration during 15 min.

The standard stock solutions gallic acid (290 mg/L), malic acid (180 mg/L), epicatechin (290 mg/L), vanillin (200 mg/L), ferulic acid (200 mg/L), luteolin (180 mg/L), salicylic acid (150 mg/L), caffeic acid (290 mg/L), protocatechuic acid (250 mg/L), quinic acid (280 mg/L) were used to quantify phenolic compounds in the extracts by comparing their retention times with the standards. The purity of the standards was 99% for gallic and salicylic acids, 95% for caffeic acid, and 90% for epicatechin, which were considered for quantification. Each sample was analyzed in triplicate.

## 4. Conclusions

Brazilian endemic flora serves as a reservoir of multiple bioactivities making it crucial to explore the potential of the pequi fruit in this context. The pequi peel presented higher levels of phenolic compounds and a higher antioxidant capacity. Epicatechin was identified as the most abundant phenolic compound, followed by caffeic, salicylic, and gallic acids. Additionally, fingerprinting revealed compounds associated with several beneficial health effects. Given the substantial amount of waste generated from pequi peel, leveraging it for the development of new products appears advantageous. This approach adds value to these wastes and agro-industrial by-products while also mitigating environmental pollution. Moreover, the results highlight pequi peel as a promising option for future applications, given its richness in natural antioxidants.

## Figures and Tables

**Table 1:** Antioxidant activity in pequi peel extracts according to the FRAP, DPPH, and ABTS methods expressed by µmol Trolox Equivalent Antioxidant Capacity (TEAC). Total phenolic content (TPC) in mg GAE/g dry mass (DM).

Sample		Assay		
*C. brasiliense* (peel)	FRAP	DPPH	ABTS	TPC
	µmol Trolox/g DM	µmol Trolox/g DM	µmol Trolox/g DM	mg GAE/g DM
	4025.17 ± 147.63	2615.47 ± 136.08	12,423.01 ± 339.22	204.77 ± 8.63

Legend: Data are expressed as the mean ± standard deviation (n = 3).

**Table 2:** Suggested compounds from pequi extract paper spray mass spectrometry (PS-MS) in positive and negative ionization mode.

*m*/*z*	Ion Type	Identification Attempt	Class	MS/MS	Reference
138	[M + H]^+^	Salicylic acid	Benzoic acid derivative	-	[29]
180	[M + H]^+^	Caffeic acid	Phenolic compounds	-	[30]
239	[M − H]^−^	Hydroxybenzyl malic acid (eucomic acid)	Benzoic acid derivative	149, 177, 179, 195, 239	[17]
255	[M − H]^−^	Pinocembrin	Flavonoid	169, 211, 255	[31]
267	[M − H]^−^	Formononetin	Flavonoid	208, 223, 267	[17]
285	[M − H]^−^	Luteolin	Flavonoid	213, 223, 239, 241, 243, 257, 267	[17]
289	[M − H]^−^	Epicatechin	Flavonoid	-	[32]
290	[M + H]^+^	Epicatechin	Flavonoid	-	[33]
301	[M + H]^+^	Chrysoeriol	Flavonoid	286, 301	[17]
301	[M − H]^−^	Ellagic acid	Benzoic acid derivative	-	[34]
307	[M + H]^+^	Galocatechin	Flavonoid	289, 307	[35]
317	[M + H]^+^	Rhamnetin	Flavonoid	165, 317	[17]
315	[M − H]^−^	Isorhamnetin	Flavonoid	300, 315	[17]
325	[M − H]^−^	Hexoside *p*-coumaric acid	Phenylpropanoid	119, 325	[17]
365	[M + H]^+^	Sucrose	Sugar	203, 365	[36]
367	[M − H]^−^	3-*O*-Feruloylquinic acid	Phenylpropanoid	149, 193, 367	[37]
431	[M − H]^−^	Isovitexin	Flavonoid	311	[34]
433	[M − H]^−^	Quercetin arabinoside	Flavonoid	300, 433	[17]
433	[M − H]^−^	Quercetin-pentosise	Flavonoid	-	[34]

**Table 3:** Concentration of phenolic compounds identified in pequi peel extracts by HPLC expressed in mg/g dry mass (DM).

Number	Compound	Concentration (mg/gDM)
1	Gallic acid	0.05 ± 0.01
2	Protocatechuic acid	<LOD
3	Caffeic acid	4.32 ± 1.63
4	Epicatechin	7.36 ± 1.85
5	Vanillin	<LOD
6	Salicylic acid	1.28 ± 0.02
7	Ferulic acid	<LOD

Legend: Data are expressed as the mean ± standard deviation (n = 3).

## Data Availability

The original contributions presented in the study are included in the article, further inquiries can be directed to the corresponding author.

## Supplementary Materials

The following supporting information can be downloaded at: https://www.mdpi.com/article/10.3390/plants13152016/s1, Figure S1: Calibration curve of DPPH; Figure S2: Calibration curve of FRAP; Figure S3: Calibration curve of ABTS; Figure S4: Calibration curve of TPC; Figure S5: PS(-)MS of the pequi peel extract; Figure S6: PS(-)MS of the pequi peel extract, ion of *m*/*z* 285 (ascribed as deprotonated Luteolin); Figure S7: PS(-)MS of the pequi peel extract, ion of *m*/*z* 301 (ascribed as deprotonated Ellagic acid); Figure S8: PS(-)MS of the pequi peel extract, ion of *m*/*z* 433 (ascribed as deprotonated Quercetin.
