# Supplementary material for: Phenolic Compounds Characterization of Caryocar brasiliense Peel with Potential Antioxidant Activity
Source: Plants (Basel). 2024 Jul 23;13(15):2016. doi: 10.3390/plants13152016 (PMC11314331; doi:10.3390/plants13152016)
Supplement: Supplementary file 1 [file plants-13-02016-s001.zip › plants-3028014-supplementary.pdf]

# Supplementary Material

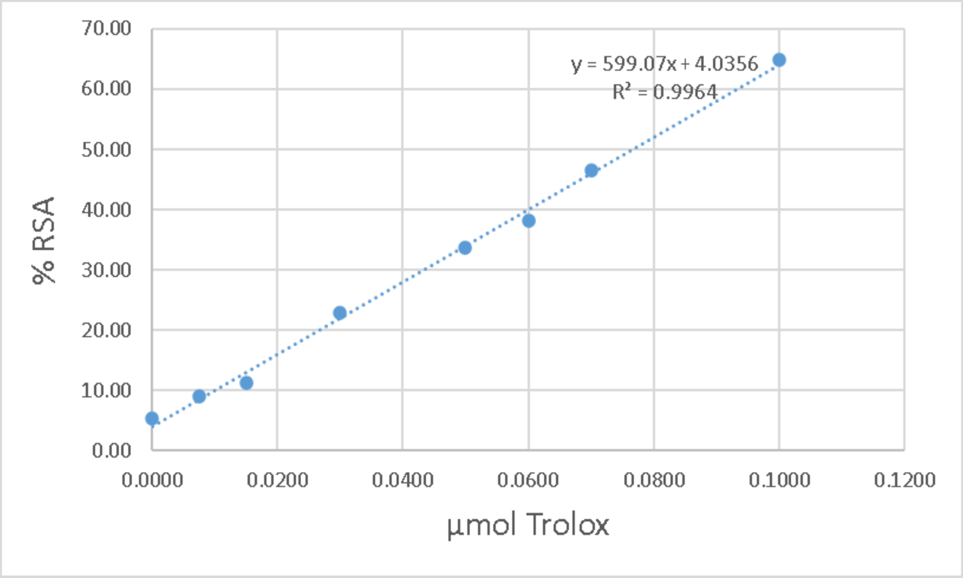


**Figure S1**. Calibration curve of DPPH.

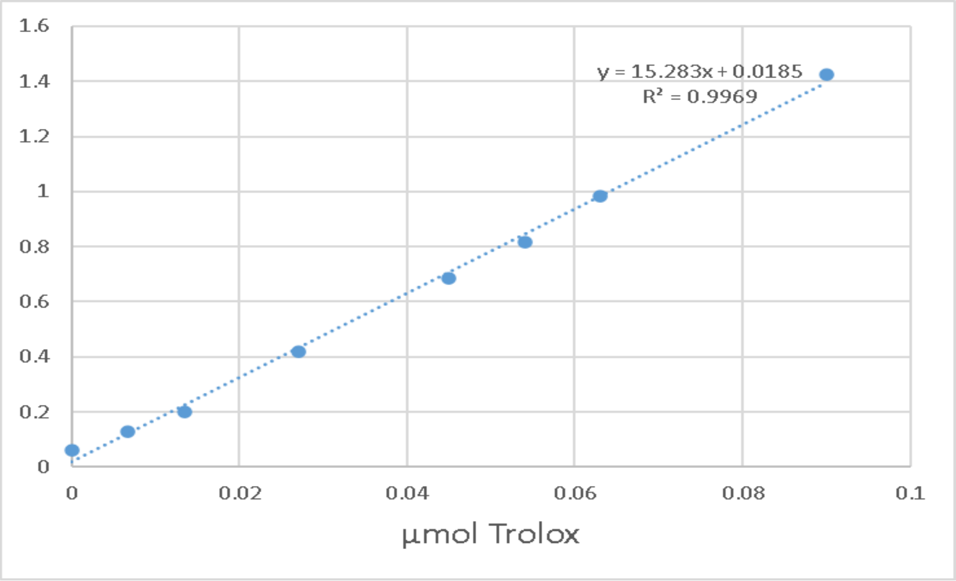


**Figure S2**. Calibration curve of FRAP

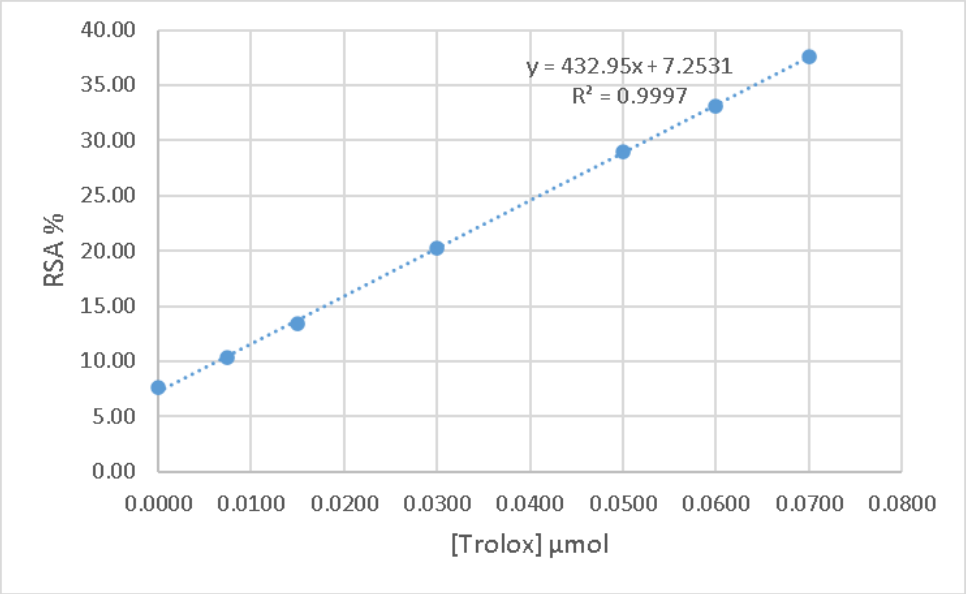


**Figure S3**. Calibration curve of ABTS.

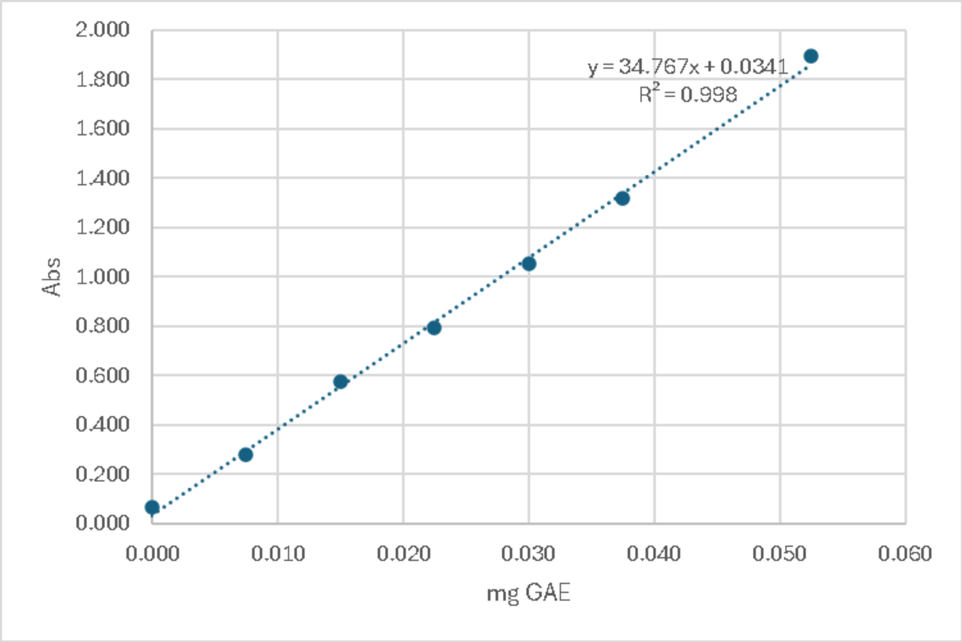


**Figure S4**. Calibration curve of TPC

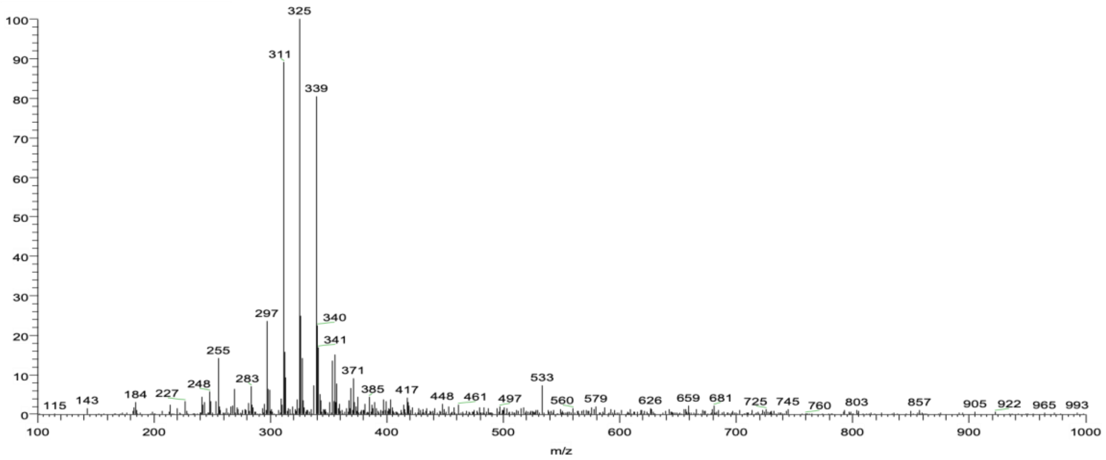


**Figure S5.** PS(-)MS of the pequi peel extract.

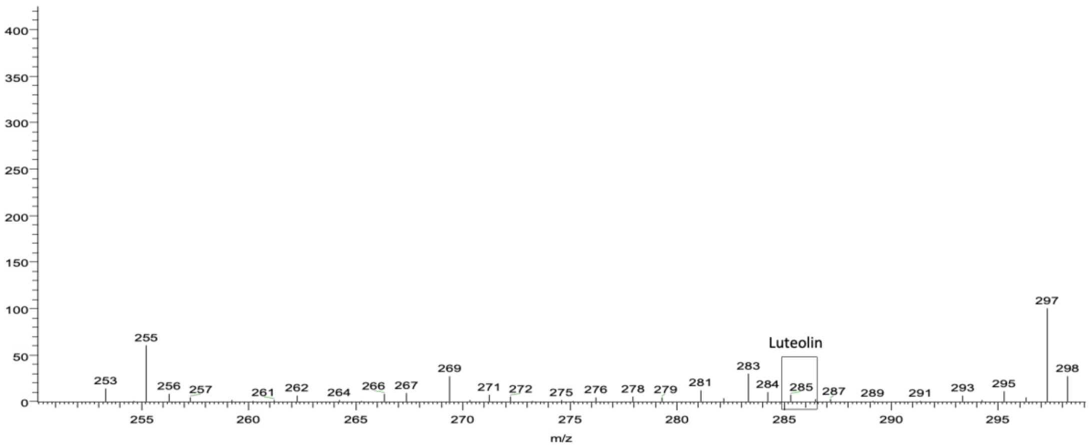


**Figure S6.** PS(-)MS of the pequi peel extract, ion of *m/z* 285 (ascribed as deprotonated Luteolin).

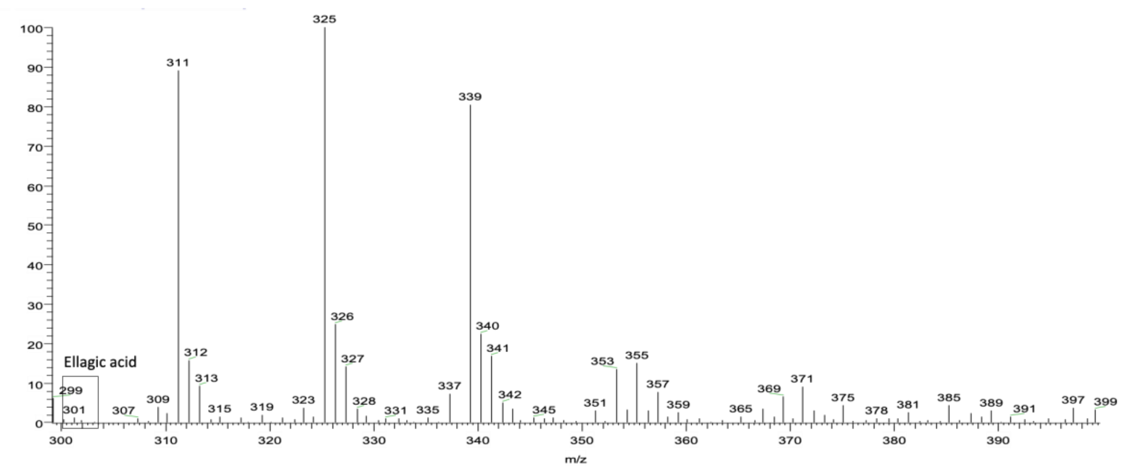


**Figure S7.** PS(-)MS of the pequi peel extract, ion of *m/z* 301 (ascribed as deprotonated Ellagic acid).

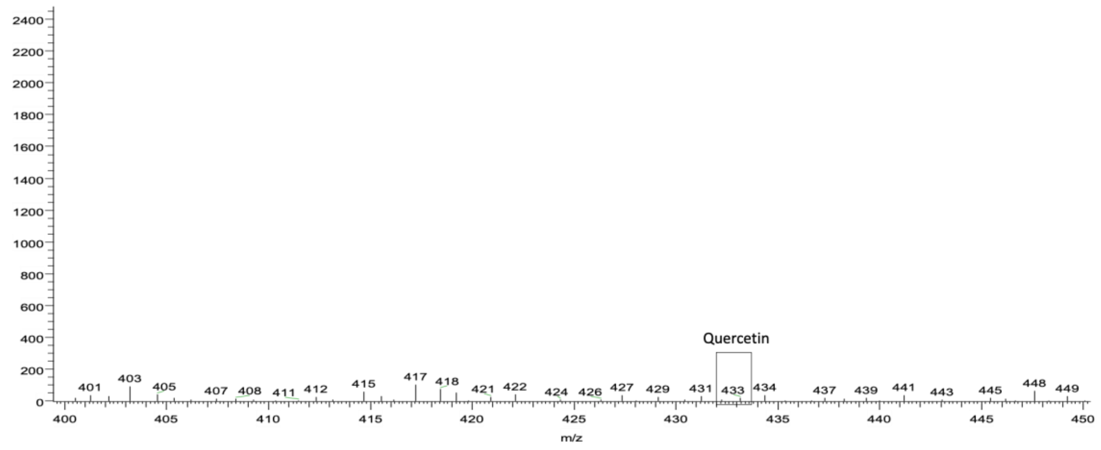


**Figure S8.** PS(-)MS of the pequi peel extract, ion of *m/z* 433 (ascribed as deprotonated Quercetin).
